# Comparison of Long-Term Clinical Outcomes of Zirconia and Lithium Disilicate Prostheses: A Retrospective Cohort Study

**DOI:** 10.3390/biomimetics10110740

**Published:** 2025-11-05

**Authors:** Basak Topdagi, Muhammed Kurum, Ceren Cakar Guler, Mohammad Abo Haoran

**Affiliations:** 1 Department of Prosthodontics, School of Dentistry, Hamidiye Campus, University of Health Sciences, 34668 Istanbul, Turkey; 2 Department of Prosthodontics, School of Dentistry, Bingol University, 12000 Bingöl, Turkey; mkurum@bingol.edu.tr (M.K.); cguler@bingol.edu.tr (C.C.G.); 3 Department of Prosthodontics, School of Dentistry, Ataturk University, 25100 Erzurum, Turkey; mhd.horan@gmail.com

**Keywords:** zirconia, lithium disilicate, prosthesis survival, dental complications, esthetics, implant-supported restorations

## Abstract

**Objectives**: This study aimed to compare the 5-year cumulative survival rates and clinical outcomes of zirconia and lithium disilicate restorations in both tooth- and implant-supported prostheses, focusing on survival, technical and biological complications, as well as patient-reported satisfaction. **Materials and Methods**: A retrospective cohort of 200 patients treated with either zirconia (n = 100) or lithium disilicate (n = 100) fixed restorations between 2020 and 2024 was analyzed. Only cases with a minimum follow-up of 5 years were included. Clinical parameters (fracture, chipping, retention loss, secondary caries, peri-implant complications), radiographic outcomes (marginal bone loss, periapical stability), and patient satisfaction (VAS scores for esthetics and function) were evaluated. Kaplan–Meier survival analysis and subgroup analyses (anterior/posterior, tooth-/implant-supported) were performed. **Results**: At 5 years, the cumulative survival rate was 94.0% for zirconia and 89.0% for lithium disilicate (*p* = 0.210). Technical complications were lower with zirconia (14.0% vs. 21.0%, *p* = 0.182), including fewer fractures (6.0% vs. 12.0%, *p* = 0.126). Chipping (5.0% vs. 7.0%) and debonding (3.0% vs. 2.0%) showed no significant differences. Biological outcomes were comparable: secondary caries (7.0% vs. 11.0%, *p* = 0.332), endodontic issues (4.0% vs. 6.0%, *p* = 0.516), peri-implant mucositis (9.0% vs. 12.0%, *p* = 0.495) and peri-implantitis (3.0% vs. 5.0%, *p* = 0.470). Radiographically, periapical stability was preserved in most cases (93.0% vs. 89.0%, *p* = 0.317), and the mean marginal bone loss was slightly lower with zirconia (0.46 ± 0.25 mm vs. 0.53 ± 0.30 mm, *p* = 0.148). Patient-reported outcomes were favorable in both groups, with esthetic VAS scores of 8.6 vs. 8.2 (*p* = 0.072) and functional scores of 8.4 vs. 8.0 (*p* = 0.085). Zirconia was rated higher in posterior/implant-supported cases, while lithium disilicate was preferred in anterior restorations. **Conclusions**: Both zirconia and lithium disilicate restorations demonstrated favorable long-term outcomes, with zirconia trending toward superior mechanical reliability in posterior and implant-supported restorations, and lithium disilicate excelling in esthetic performance, particularly in anterior regions. Material selection should be guided by clinical indication, occlusal load distribution, and esthetic requirements.

## 1. Introduction

The replacement of missing teeth with fixed prosthetic restorations plays a critical role in restoring both esthetics and function in dentistry. Over the past few decades, the development of ceramic-based materials has significantly influenced prosthodontic practice, largely due to their biocompatibility, mechanical strength, and superior esthetic qualities compared to traditional metal-ceramic restorations. Recent advances in ceramic nanostructure design and surface engineering have further improved translucency and bonding strength in zirconia-based systems [1,2,3,4]. As a result, ceramics have become increasingly preferred as restorative materials in clinical settings [5,6,7]. Among the wide range of available ceramics, zirconia and lithium disilicate are the most widely used due to their favorable mechanical and optical properties, though they differ substantially in performance under long-term clinical conditions [8,9,10].

Zirconia (ZrO_2_)-based restorations are widely utilized, particularly in posterior regions, because of their excellent mechanical strength (flexural strength of approximately 900–1200 MPa), low water solubility, and outstanding biocompatibility [11,12]. These features provide resistance to fracture and durability under high occlusal forces. However, conventional full-contour zirconia exhibits limited translucency, which can restrict its use in highly esthetic zones such as the anterior region. To address this limitation, newer generations of translucent zirconia have been introduced, offering improved esthetic performance at the expense of a modest reduction in mechanical strength [3,13].

In recent years, zirconia and lithium disilicate have become dominant materials in fixed prosthodontics. According to recent clinical surveys and market analyses, zirconia-based restorations represent approximately 60–70% of all-ceramic fixed dental prostheses worldwide, owing to their superior strength and long-term reliability, particularly in posterior and implant-supported cases [3,13,14]. In contrast, lithium disilicate accounts for about 20–30% of all-ceramic restorations, being preferred in anterior regions for its esthetic translucency and bonding capability with adhesive cementation [1,14,15]. Clinically, zirconia is indicated for high-load situations, while lithium disilicate is chosen for minimally invasive veneers, inlays, and anterior crowns where optical integration is critical. The balance between mechanical durability and esthetics therefore continues to guide material selection in modern prosthodontic practice.

Lithium disilicate ceramics, on the other hand, are known for their excellent esthetic outcomes, with flexural strengths ranging from 360 to 450 MPa [1,14,15,16]. Their high translucency and optical characteristics closely mimic natural dentition, making them the material of choice for anterior restorations where esthetics are paramount. Despite these advantages, the comparatively lower mechanical resistance of lithium disilicate raises concerns regarding their long-term survival in posterior restorations, where occlusal forces are considerably greater [4,17].

The evaluation of prosthetic success involves multiple clinical parameters, including marginal adaptation, biomechanical durability, chewing function, complication rates, and patient satisfaction [18,19]. Although both zirconia and lithium disilicate ceramics are established in restorative practice, studies directly comparing their long-term outcomes are relatively limited. Moreover, the available literature often reports variable findings, creating uncertainty about the superiority of one material over the other in terms of clinical longevity and esthetic performance [2,20,21].

Given these considerations, further research is warranted to provide clinicians with evidence-based guidance on material selection. In this study, we aimed to compare the long-term success rates of zirconia and lithium disilicate fixed prostheses. By assessing both clinical and radiographic outcomes, including mechanical survival, marginal adaptation, esthetic satisfaction, and biological complications, this study seeks to clarify the advantages and limitations of each material in implant-supported and tooth-supported prostheses. The results are expected to contribute to informed clinical decision-making and optimized patient outcomes in prosthodontics.

## 2. Materials and Methods

### 2.1. Study Design and Patient Selection

This study was reviewed and approved by the Ethics Committee of Hamidiye Scientific Research Board, University of Health Sciences (Approval no: 10/41, Date: 8 May 2025). All procedures were conducted in accordance with the principles of the Declaration of Helsinki. This study was designed as a multicenter, retrospective, observational cohort study. It was conducted at the Department of Prosthodontics, Faculty of Dentistry, University of Health Sciences, and at the Bingöl Oral and Dental Health Center. The study protocol was approved by the local institutional review boards of the participating institutions and adhered to the ethical principles outlined in the Declaration of Helsinki. All patient data were anonymized prior to analysis and were obtained from electronic health records and patient files.

The study population consisted of patients who had received fixed prosthetic treatment with either zirconia or lithium disilicate restorations between January 2020 and December 2024. Only patients with a minimum postoperative follow-up period of five years and complete clinical documentation were included. Only patients with restorations in function for at least five years were included. Cases with less than one year of prosthesis function were excluded to avoid early postoperative bias and to allow for evaluation of long-term biological outcomes. Patients were eligible for inclusion if they had been treated with zirconia- or lithium disilicate-based fixed prostheses between January 2020 and December 2024, with a minimum follow-up period of five years. Only cases performed according to standardized prosthetic protocols and supported by complete clinical and radiographic documentation throughout the follow-up period were considered. Exclusion criteria included patients with severe malocclusion, those presenting with parafunctional habits such as severe bruxism or nail biting, individuals with systemic diseases leading to advanced bone loss, and patients with incomplete records or insufficient follow-up of less than five years.

The sample size was determined using G*Power (version 3.1.9.2, Universität Düsseldorf, Germany) based on a medium effect size (f = 0.25) according to Cohen’s conventions, with α = 0.05 and power (1–β) = 0.80, resulting in a required minimum of 100 patients per group. This calculation was based on the primary endpoint (5-year survival rate). To reduce the likelihood of type I error due to multiple subgroup comparisons, a Bonferroni-adjusted significance threshold (αadj = 0.0125) was applied for secondary analyses.

### 2.2. Data Collection and Variables

Demographic information, treatment procedures, prosthesis characteristics, complication rates, and long-term clinical outcomes were retrieved from patient files and digital records. Cementation protocols and preparation designs were standardized across all participating centers. In this study, the term fixed dental prosthesis (FDP) refers to multi-unit bridge restorations replacing one or more missing teeth, while single crowns denote single-unit fixed restorations. This classification was adopted to ensure consistency and clarity in reporting the clinical outcomes of each restoration type. Monolithic zirconia restorations were produced using 4Y-PSZ (Katana Zirconia STML, Kuraray Noritake Dental Inc., Tokyo, Japan; Batch No. KZST-2411), whereas veneered zirconia restorations were fabricated from 3Y-TZP (IPS e.max ZirCAD, Ivoclar Vivadent, Schaan, Liechtenstein; Batch No. ZCAD-2409). Lithium disilicate restorations were made from IPS e.max CAD (Ivoclar Vivadent; Batch No. EMC-2405). All zirconia restorations were sintered at 1500 °C for 2 h according to the manufacturer’s recommendations. The following parameters were systematically evaluated:

➢ **Biomechanical performance:** Assessed by incidence of fractures and cracks.
➢ **Marginal adaptation:** Evaluated in terms of cement dissolution, marginal discrepancies, and occurrence of secondary caries.
➢ **Chewing function and patient satisfaction:** Assessed using a Visual Analog Scale (VAS) ranging from 0 (not satisfied) to 10 (fully satisfied). The VAS method followed previously validated protocols for assessing esthetic and functional satisfaction in prosthodontic research, as described by Chander, Patil, and Tosun et al. [22,23,24].
➢ **Radiographic evaluation:** Included assessment of peri-apical conditions for tooth-supported restorations and peri-implant bone stability for implant-supported restorations. In this study, periapical status in tooth-supported restorations was assessed to detect apical or periodontal pathology (not bone loss), as this parameter best represents the biological integrity of natural teeth. For implant-supported prostheses, marginal bone loss was measured as the vertical distance from the implant shoulder (IS) to the first bone-to-implant contact (BIC) on mesial and distal aspects. This distinction is anatomically justified and aligns with radiographic protocols cited in Rehberger Bescos et al. and Weigel et al. [25,26]. All implants were placed at the crestal level following a standard two-stage protocol, ensuring uniform bone reference points for radiographic assessment. Radiographic evaluations were performed using standardized periapical radiographs obtained with the paralleling technique and digital sensors (Planmeca ProX, Helsinki, Finland). Measurements of marginal bone levels and periapical status were conducted with ImageJ software (version 1.53, NIH, Bethesda, MD, USA). All radiographs were analyzed by two calibrated examiners, and intra-examiner reliability showed excellent agreement (ICC = 0.94). Identical imaging and measurement protocols were used for both tooth- and implant-supported prostheses, ensuring comparability between zirconia and lithium disilicate groups. For implant-supported prostheses, marginal bone loss was defined as the vertical distance between the implant shoulder and the most coronal bone-to-implant contact on the mesial and distal surfaces, following the standardized radiographic protocol described by Rehberger Bescos et al. [25]. For implant-supported restorations, marginal bone loss was measured as the vertical distance from the implant shoulder (IS) to the first bone-to-implant contact (BIC). For tooth-supported restorations, the vertical distance from the cementoenamel junction (CEJ) to the alveolar crest (AC) was measured to evaluate crestal bone integrity. All measurements were performed using ImageJ software (version 1.53, NIH, USA), calibrated according to sensor pixel dimensions. Standardized periapical radiographs were obtained with the paralleling technique using a fixed film holder to minimize distortion. Two experienced examiners performed all measurements, and intra- and inter-examiner reliability was excellent (ICC = 0.94 and 0.91, respectively). These protocols ensured reproducible and comparable radiographic assessments across zirconia and lithium disilicate groups. Radiographic evaluation protocols (measurement landmarks, calibration, distortion control) were adapted from Mailoa et al., who defined distances from the cementoenamel junction (CEJ), implant platform, and the first bone-to-implant contact [27]. In similar studies, CBCT-based measurements have validated periapical radiograph-based assessments of marginal bone loss [26,28].
➢ **Complications:** biological and technical complications, including chipping, debonding, and loss of retention, were recorded.

### 2.3. Clinical Outcomes

The primary outcome was long-term prosthesis survival, defined as the restoration remaining in situ without catastrophic failure (fracture or loss requiring replacement). The secondary outcomes included complication rates, marginal adaptation, patient satisfaction scores, and esthetic performance.

### 2.4. Statistical Analysis

Statistical analyses were performed using IBM SPSS Statistics software (version 27.0, IBM Corp., Armonk, NY, USA). Continuous variables were expressed as mean ± standard deviation (SD), while categorical variables were presented as frequencies and percentages. The chi-square test was applied for categorical comparisons, and independent-samples *t* tests were used for continuous variables. Long-term survival rates of zirconia and lithium disilicate prostheses were analyzed using Kaplan–Meier survival curves. Survival probabilities were estimated using the Kaplan–Meier method, and differences between groups were compared using the log-rank test. To account for potential competing risks (e.g., patient loss to follow-up or prosthesis replacement for unrelated reasons), cumulative incidence functions (CIFs) were also computed using the Fine and Gray model. Censoring was handled non-informatively, assuming random loss to follow-up. Additionally, a Cox proportional hazards model was used to assess time-dependent covariates and to verify proportionality assumptions. For subgroup comparisons (tooth-/implant-supported, anterior/posterior), Bonferroni-adjusted *p*-values (αadj = 0.0125) were applied to control for multiple testing, and post hoc power was evaluated to ensure adequate sensitivity. A *p*-value < 0.05 was considered statistically significant.

## 3. Results

**Figure 1:**
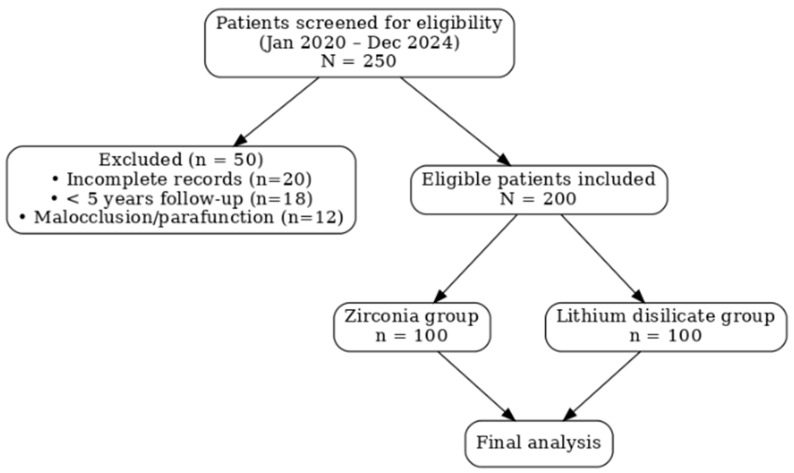
Flow diagram showing patient selection and allocation to zirconia and lithium disilicate groups.

Between January 2020 and December 2024, 250 patients were screened for eligibility. Of these, 50 patients were excluded due to incomplete records (n = 20), insufficient follow-up of less than five years (n = 18), or the presence of severe malocclusion and parafunctional habits such as bruxism (n = 12). A total of 200 patients met the inclusion criteria and were analyzed: zirconia group (n = 100) and lithium disilicate group (n = 100). The mean follow-up period was 5.3 ± 0.7 years (range 5.0–6.5 years) (Figure 1).

Baseline demographic and clinical characteristics were comparable between the zirconia and lithium disilicate groups (Table 1). The mean age was 51.8 ± 10.2 years in the zirconia group and 50.7 ± 9.8 years in the lithium disilicate group (*p* = 0.420). The proportion of females was 62.0% in the zirconia group and 59.0% in the lithium disilicate group (*p* = 0.680). The mean follow-up period was 5.4 ± 0.6 years for zirconia and 5.3 ± 0.7 years for lithium disilicate restorations (*p* = 0.550). With respect to type of support, 58 zirconia restorations (58%) and 61 lithium disilicate restorations (61%) were tooth-supported, whereas 42 zirconia (42%) and 39 lithium disilicate (39%) were implant-supported (*p* = 0.700). Regarding location, 40 zirconia restorations (40%) and 44 lithium disilicate restorations (44%) were placed in the anterior region, while 60 zirconia (60%) and 56 lithium disilicate (56%) were located posteriorly (*p* = 0.560). In terms of type of restoration, 72 zirconia restorations (72%) and 75 lithium disilicate restorations (75%) were single crowns, whereas 28 zirconia (28%) and 25 lithium disilicate (25%) were fixed dental prostheses (FDPs) (*p* = 0.640) (Table 1).

The majority of zirconia restorations were monolithic (68%), while 32% were veneered. In contrast, lithium disilicate restorations were predominantly veneered (61%), with 39% monolithic (*p* = 0.032). Unit distribution revealed that 72% of zirconia restorations were single crowns and 28% were fixed dental prostheses (FDPs), compared to 75% single crowns and 25% FDPs in the lithium disilicate group (*p* = 0.640).

**Figure 2:**
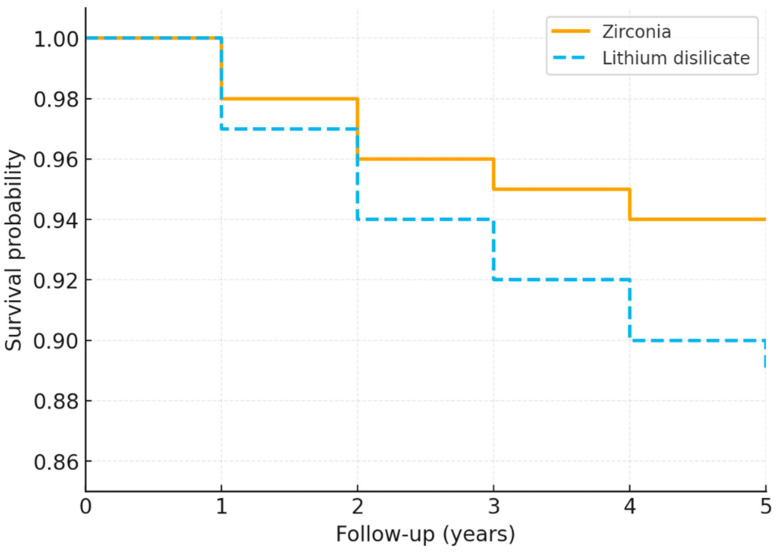
Kaplan–Meier survival curves for zirconia and lithium disilicate prostheses over a 5-year follow-up period.

At 5 years, the estimated survival rate was 94.0% for zirconia and 89.0% for lithium disilicate (*p* = 0.210). Kaplan–Meier survival analysis demonstrated a trend toward higher survival in the zirconia group, although the difference did not reach statistical significance (Figure 2). The median cumulative survival rate was not reached in either group during the observation period.

Overall technical complication rates were 14.0% in the zirconia group compared with 21.0% in the lithium disilicate group (*p* = 0.182). Fracture or catastrophic failure occurred in 6 patients (6.0%) with zirconia restorations and 12 patients (12.0%) with lithium disilicate restorations (*p* = 0.126). Veneer chipping was observed in 5 patients (5.0%) in the zirconia group versus 7 patients (7.0%) in the lithium disilicate group (*p* = 0.552). Loss of retention or debonding was reported in 3 cases (3.0%) for zirconia and 2 cases (2.0%) for lithium disilicate (*p* = 0.652) (Table 2).

In tooth-supported restorations, secondary caries occurred in 7 patients (7.0%) in the zirconia group and 11 patients (11.0%) in the lithium disilicate group (*p* = 0.332). Endodontic complications were recorded in 4 patients (4.0%) with zirconia and 6 patients (6.0%) with lithium disilicate restorations (*p* = 0.516). For implant-supported restorations, peri-implant mucositis was observed in 9 patients (9.0%) with zirconia and 12 patients (12.0%) with lithium disilicate prostheses (*p* = 0.495). Peri-implantitis was reported in 3 cases (3.0%) in the zirconia group compared to 5 cases (5.0%) in the lithium disilicate group (*p* = 0.470). Radiographic evaluation revealed stable periapical status in 93 patients (93.0%) with zirconia and 89 patients (89.0%) with lithium disilicate restorations (*p* = 0.317). Mean marginal bone loss was 0.46 ± 0.25 mm for zirconia and 0.53 ± 0.30 mm for lithium disilicate restorations, with no significant difference between groups (*p* = 0.148) (Table 3).

Evidence of cement washout or marginal discrepancy was observed in 8 patients (8.0%) in the zirconia group compared with 12 patients (12.0%) in the lithium disilicate group (*p* = 0.340).

**Figure 3:**
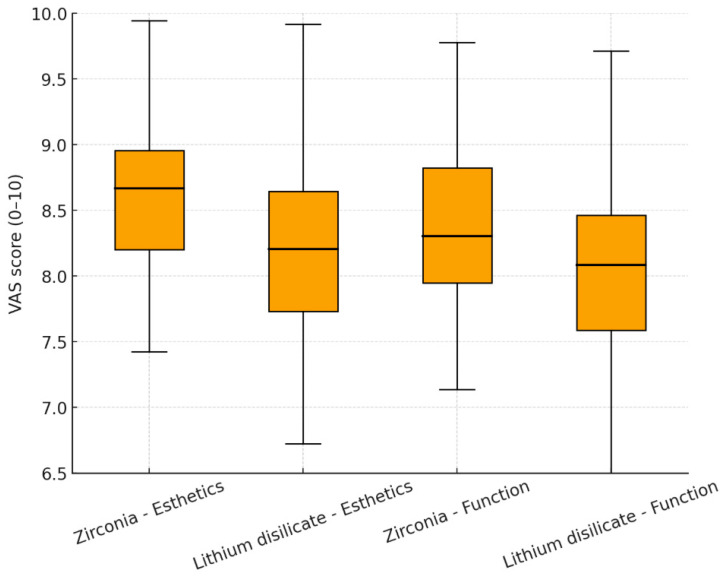
Visual Analog Scale (VAS) scores for esthetics and function in zirconia and lithium disilicate prostheses.

VAS satisfaction scores for esthetics were 8.6 ± 0.6 for zirconia and 8.2 ± 0.7 for lithium disilicate restorations (*p* = 0.072). For function and chewing ability, mean VAS scores were 8.4 ± 0.7 in the zirconia group and 8.0 ± 0.8 in the lithium disilicate group (*p* = 0.085) (Figure 3).

For tooth-supported restorations, periapical status remained stable in 93.0% of zirconia cases compared with 89.0% of lithium disilicate cases (*p* = 0.317). For implant-supported prostheses, the mean radiographic marginal bone loss at the final follow-up was 0.46 ± 0.25 mm in the zirconia group and 0.53 ± 0.30 mm in the lithium disilicate group (*p* = 0.148).

When stratified by type of support, the 5-year cumulative survival rate for implant-supported prostheses was 93.0% in the zirconia group compared with 88.0% in the lithium disilicate group (*p* = 0.224). For tooth-supported prostheses, the cumulative survival rate was 95.0% for zirconia and 90.0% for lithium disilicate restorations (*p* = 0.210). By location, in the posterior region, zirconia restorations demonstrated a survival rate of 93.0% compared with 87.0% for lithium disilicate (*p* = 0.198). In the anterior region, survival rates were slightly higher overall, with 95.0% in zirconia and 91.0% in lithium disilicate restorations (*p* = 0.245). These subgroup analyses indicate that zirconia restorations tended to perform better in both posterior and implant-supported situations, while lithium disilicate restorations showed comparable survival in anterior and tooth-supported cases. However, none of the observed differences reached statistical significance (Table 4).

## 4. Discussion

The present retrospective cohort study compared the 5-year clinical outcomes of zirconia and lithium disilicate restorations in both tooth- and implant-supported prostheses. Our findings demonstrated comparable survival rates between the two ceramic systems, with zirconia showing a slightly higher, though statistically non-significant, cumulative survival rate (94.0% vs. 89.0%). Additionally, zirconia restorations exhibited fewer technical and biological complications, particularly in posterior and implant-supported cases, whereas lithium disilicate restorations provided esthetic outcomes and patient satisfaction that were not inferior to zirconia.

Our results align with the observations of Heintze and Rousson, who in a systematic review reported survival rates of zirconia-supported fixed prostheses ranging from 90 to 95% over 5 years, emphasizing their reliability under high occlusal forces [18]. Belli et al. highlighted the superior fracture resistance and longer lifetime estimation of CAD/CAM zirconia-based restorations compared to glass-ceramics [12]. These outcomes support our finding of lower fracture incidence in zirconia restorations (6.0%) compared to lithium disilicate (12.0%) (Table 2) supports their superior mechanical durability.

In contrast, lithium disilicate restorations are known for their excellent esthetic qualities. Floriani et al. and Jurado et al. demonstrated the optical superiority and natural tooth-like translucency of lithium disilicate, which accounts for their preference in anterior restorations [15,16]. Our results confirm these findings, as patient satisfaction for esthetics was high in both groups but slightly higher for lithium disilicate in anterior placements.

Chipping rates observed in our zirconia group (5.0%) were similar to those reported by Aboushelib et al., who noted that veneered zirconia restorations are more prone to veneer fractures due to mismatches in thermal expansion coefficients [10]. However, the predominance of monolithic zirconia in our sample may explain the relatively low chipping rate, consistent with the clinical report by Linkevicius, who emphasized the improved longevity of monolithic zirconia restorations in posterior regions [11]. Recent advancements in ceramic material technology, particularly the development of highly translucent 4Y-PSZ and 5Y-PSZ zirconia, have significantly improved the balance between mechanical strength and optical performance. Modern surface engineering methods, including selective infiltration etching and CAD/CAM-based finishing protocols, have further enhanced adhesion durability and resistance to fatigue and chipping [1,2,3,4]. These innovations provide a scientific rationale for the superior longevity and clinical reliability observed in monolithic zirconia restorations.

Regarding biological complications, our findings of marginal bone loss (0.46 mm for zirconia vs. 0.53 mm for lithium disilicate) are in accordance with the results of Edelhoff et al., who reported minimal radiographic differences between ceramic systems over 5 years [19]. Furthermore, the incidence of peri-implant mucositis and peri-implantitis was similar across groups, echoing the conclusions of Nishihara et al., who underscored the biocompatibility of both zirconia and lithium disilicate implant-supported prostheses [5]. The influence of prosthesis age on biological complications has been previously recognized. However, as all patients in our cohort had restorations functioning for a minimum of five years, early complications related to recent placement were inherently excluded. The comparable rates of peri-implant mucositis and secondary caries observed in this study therefore represent long-term biological stability rather than early adaptation effects. Within the 5-year observation window (mean 5.3 ± 0.7 years), no progressive increase in marginal bone loss was detected among patients with longer functional duration. This suggests that both zirconia and lithium disilicate restorations maintained stable peri-implant bone levels throughout the mid-term follow-up period.

In addition to biocompatibility, corrosion resistance plays a crucial role in the long-term clinical success of ceramic restorations. Zirconia and lithium disilicate both exhibit excellent electrochemical stability and chemical inertness, minimizing ion release and surface degradation in the oral environment. Surface roughness is another critical factor influencing bacterial adhesion and biofilm formation. Highly polished and glazed zirconia surfaces have been shown to significantly reduce bacterial colonization compared to roughened or veneered surfaces, thereby lowering the risk of peri-implant inflammation and secondary caries. Optimizing surface topography and maintaining corrosion-resistant interfaces are therefore essential for preventing biofilm accumulation and ensuring long-term biological stability of both materials [29,30,31,32,33]. In this study, periapical status was evaluated in tooth-supported restorations to identify any apical or periodontal pathology that could compromise the biological integrity of the natural tooth, whereas marginal bone loss was analyzed in implant-supported prostheses as the principal radiographic indicator of peri-implant tissue health and osseointegration stability. These distinct parameters were therefore selected according to their anatomical and clinical relevance.

The comparable overall survival and mechanical/biological complication profiles of zirconia and lithium disilicate over 5 years in our study are consistent with the comprehensive meta-analysis by Pjetursson et al., which reported high short- to mid-term success rates of all-ceramic implant-supported single crowns and highlighted a higher incidence of chipping in veneered designs [34]. This analysis emphasized that monolithic designs are significantly more resistant to chipping; in line with this, the predominance of monolithic zirconia in our cohort explains the relatively low chipping rate observed.

Randomized evidence also supports the notion that material-related differences in implant-supported single crowns may be clinically minimal in the early period. In a multicenter RCT with 1-year follow-up, Strasding et al. reported 100% survival of micro-veneered lithium disilicate (LDS) and zirconia crowns, with minor chipping occurring only in the LDS group and none in zirconia [35]. This finding is consistent with our observation that zirconia restorations tended to show lower technical complication rates in the implant and posterior subgroups.

The superiority of monolithic zirconia (MZ) over porcelain-veneered zirconia (PVZ) has also been systematically demonstrated in tooth-supported fixed dental prostheses. Shihabi et al., in a meta-analysis, reported that both minor and major chipping were significantly lower in MZ compared to PVZ at 5 years, and overall failure rates were more favorable for MZ [36]. The clinical rationale behind this difference lies in the bilayer structure’s susceptibility to veneer chipping due to thermal expansion mismatch and residual stresses; in our series, the relatively low proportion of veneered restorations likely explains the limited incidence of chipping.

From a long-term perspective, the prospective 15-year observational study by Khijmatgar et al. demonstrated that cumulative failures in zirconia-based crowns and FDPs become more evident as the time horizon extends, with failure rates increasing notably beyond the 5-year period [37]. This supports the interpretation that our 5-year results reflect at best a medium-term performance, and that longer follow-up may reveal higher rates of technical issues, particularly retention loss and chipping. Similarly, marginal bone changes over the long term were contextualized with reference to Weigel et al. who reported peri-implant bone level changes adjacent to natural teeth over more than 10 years, supporting the stability trends observed in our 5-year radiographic outcomes [26].

With regard to LDS, the importance of indication and design sensitivity has been clearly demonstrated in the literature. Klein et al., in a meta-analysis of veneers with over 10 years of follow-up, reported high survival and low complication rates for LDS in adhesive, partial-coverage anterior indications [38]. A multicenter practice-based study of 765 posterior partial-coverage LDS restorations reported a 5-year survival rate of 99.6% [39]. In contrast, Rinke et al., in a 5-year clinical study of ZLS/LDS-derived partial crowns, emphasized that material thickness is critical, with significant increases in fractures observed when thickness fell below 0.75–1.0 mm [40]. These findings parallel our observations, suggesting that the higher rate of fractures/complications in posterior LDS restorations may be related to high occlusal loading and/or inadequate thickness or support [38,39,40].

Finally, additional evidence strengthens clinical recommendations regarding material selection. Tajti et al. demonstrated in a systematic review and meta-analysis that monolithic zirconia represents a valid alternative to metal-ceramic restorations in posterior regions, reporting fewer chipping events in the short to mid-term [41]. In line with this, our 5-year results showing zirconia’s trend toward better performance in posterior and implant-supported subgroups reflect the clinical applicability of the above meta-analyses and RCT findings [36,41,42,43,44].

From a clinical perspective, the current findings suggest that zirconia remains the material of choice for posterior and implant-supported restorations due to its superior mechanical reliability, while lithium disilicate continues to be favored in anterior restorations where esthetics are paramount. The comparable overall survival rates indicate that both materials are viable long-term options, provided that case selection considers occlusal load distribution and esthetic requirements. It should be noted that several observed differences between zirconia and lithium disilicate did not reach statistical significance. Therefore, these findings represent trends rather than confirmatory evidence and should be interpreted with caution given the limited power of subgroup analyses.

Unlike many previously published studies that focused on either zirconia or lithium disilicate in isolation, or evaluated outcomes in short- to mid-term follow-up periods, our study directly compared the two materials in a relatively large cohort with a standardized minimum follow-up of five years. For instance, Belli et al. primarily assessed fracture resistance of CAD/CAM ceramics under laboratory conditions rather than in vivo [12], while Floriani et al. and Jurado et al. emphasized esthetic and optical properties of lithium disilicate without long-term survival data [16,18]. Similarly, systematic reviews such as that of Heintze and Rousson reported survival rates but often combined zirconia with metal-supported prostheses, limiting direct material-to-material comparisons [18]. In contrast, our work provides head-to-head clinical evidence, integrating both mechanical and esthetic outcomes across tooth- and implant-supported restorations, thereby filling an important gap in the literature.

### Limitations of the Study

A major strength of this study is the inclusion of a large cohort with a minimum of 5 years of follow-up, offering valuable insights into long-term outcomes. Standardized protocols across two clinical centers also increase the reliability of our findings. However, the retrospective design introduces inherent limitations, including possible selection bias and the lack of randomization. Furthermore, subgroup analyses indicated trends favoring zirconia in high-stress clinical situations, though the differences did not reach statistical significance, highlighting the need for larger prospective trials. Although Kaplan–Meier analysis was primarily used, a competing risk model (Fine and Gray) was additionally performed to verify the robustness of survival estimates under possible censoring and non-failure events. Another limitation of this study is the lack of surface characterization using techniques such as scanning electron microscopy (SEM), energy-dispersive X-ray spectroscopy (EDS), or contact angle measurements. As the study was retrospective and based on in situ restorations, these analyses could not be conducted without damaging the specimens. Future prospective and in vitro investigations are warranted to correlate surface morphology, elemental composition, and wettability with clinical performance.

## 5. Conclusions

In conclusion, zirconia and lithium disilicate restorations both demonstrated favorable long-term outcomes, with zirconia showing a trend toward fewer mechanical complications and lithium disilicate maintaining strong esthetic performance. These results support material selection based on individual clinical indications: zirconia for posterior and implant-supported prostheses and lithium disilicate for anterior regions where esthetics are critical. Although zirconia showed slightly fewer mechanical complications and lithium disilicate demonstrated superior esthetics, these differences were not statistically significant and should not be overinterpreted. Larger, adequately powered prospective studies are warranted to confirm these preliminary observations. Future randomized controlled studies with longer follow-up periods are warranted to further validate these findings.

## Figures and Tables

**Table 1:** Baseline demographics and clinical characteristics of patients treated with zirconia and lithium disilicate prostheses.

Characteristic	Zirconia (n = 100)	Lithium Disilicate (n = 100)	*p*-Value *
Age (years), mean ± SD	51.8 ± 10.2	50.7 ± 9.8	0.420
Sex, female, n (%)	62 (62%)	59 (59%)	0.680
Follow-up period (years), mean ± SD	5.4 ± 0.6	5.3 ± 0.7	0.550
Type of support, n (%)			
—Tooth-supported	58 (58%)	61 (61%)	0.700
—Implant-supported	42 (42%)	39 (39%)	
Location, n (%)			
—Anterior	40 (40%)	44 (44%)	0.560
—Posterior	60 (60%)	56 (56%)	
Type of restoration, n (%)			
—Single crowns	72 (72%)	75 (75%)	0.640
—Fixed dental prostheses (FDP)	28 (28%)	25 (25%)	

*: Independent-samples *t*-test for continuous variables; Chi-square (χ^2^) test for categorical variables.

**Table 2:** Technical complications by material (n, %).

Complication Type	Zirconia (n = 100)	Lithium Disilicate (n = 100)	*p*-Value *
Any technical complication	14 (14.0%)	21 (21.0%)	0.182
Fracture/catastrophic failure	6 (6.0%)	12 (12.0%)	0.126
Veneer chipping	5 (5.0%)	7 (7.0%)	0.552
Loss of retention/debonding	3 (3.0%)	2 (2.0%)	0.652

*: Chi-square (χ^2^) test.

**Table 3:** Biological complications and radiographic outcomes (n, %, and bone loss in mm).

Parameter	Zirconia (n = 100)	Lithium Disilicate (n = 100)	*p*-Value *
**Tooth-supported restorations**			
Secondary caries, n (%)	7 (7.0%)	11 (11.0%)	0.332
Endodontic complications, n (%)	4 (4.0%)	6 (6.0%)	0.516
**Implant-supported restorations**			
Peri-implant mucositis, n (%)	9 (9.0%)	12 (12.0%)	0.495
Peri-implantitis, n (%)	3 (3.0%)	5 (5.0%)	0.470
Radiographic outcomes			
Stable periapical status, n (%)	93 (93.0%)	89 (89.0%)	0.317
Marginal bone loss (mm), mean ± SD	0.46 ± 0.25	0.53 ± 0.30	0.148

*: Independent-samples *t*-test for continuous data; Chi-square (χ^2^) test for categorical data.

**Table 4:** Five-year cumulative survival rates of zirconia vs. lithium disilicate prostheses in subgroup analyses.

Subgroup	Zirconia (%)	Lithium Disilicate (%)	*p*-Value
Implant-supported	93.0	88.0	0.224
Tooth-supported	95.0	90.0	0.210
Posterior region	93.0	87.0	0.198
Anterior region	95.0	91.0	0.245

## Data Availability

The original contributions presented in the study are included in the article, further inquiries can be directed to the corresponding author.
